# Problematic Media Use(PMU) and its socio-demographic correlates among children attending a children’s tertiary care centre in Sri Lanka

**DOI:** 10.1192/j.eurpsy.2026.10547

**Published:** 2026-07-31

**Authors:** P. Vidyatilake, G. Wijetunge

**Affiliations:** Lady Ridgeway Hospital for Children, Colombo, Sri Lanka

## Abstract

**Introduction:**

Problematic media use (PMU) is increasingly recognized as a behavioral concern in children, often linked to behavioral management and a lack of alternative activities. Existing research largely derives from high-income countries, leaving significant gaps in low- and middle-income contexts. Sri Lanka, a lower-middle-income country (LMIC) with expanding digital access but restricted recreational resources, offers a distinctive setting to examine PMU. Understanding these patterns is essential for developing culturally relevant and context-specific interventions

**Objectives:**

This study aimed to compare PMU between children with psychiatric illnesses and their peers without psychiatric diagnoses, and to examine sociodemographic correlates of PMU, including parental education and household income.

**Methods:**

A cross-sectional comparative study was conducted at Lady Ridgeway Hospital, Colombo, in 2023. Participants were children aged 4–11 years: the study group comprised those attending Child and Adolescent Mental Health Services (CAMHS), while age- and sex-matched children from the Outpatient Department (OPD) served as controls. PMU was assessed using the Problematic Media Use Measure (PMUM), alongside a parent-completed sociodemographic questionnaire. Exclusion criteria included severe medical comorbidity, intellectual disability, or lack of parental consent. Written informed consent was obtained in the two local languages, and ethical approval was secured from institutional review boards.

**Results:**

The sociodemographic profiles of the two groups were broadly comparable, with no significant differences in age, gender, ethnicity, parental education, or household income. The study group (n = 214) included 120 children with ADHD (56%), 50 with Autism Spectrum Disorder (23.4%), 25 with anxiety disorders (11.7%), and 19 with depression (8.9%). Treatment modalities included medication only (60.7%), behavioural therapy only (23.4%), and combined treatment (15.9%). The comparison group comprised children without psychiatric diagnoses (n = 200). The mean PMUM score in the study group was significantly higher (23.5 ± 5.8) than in the comparison group (18.2 ± 4.3). Children with ADHD lower household income and lower parental education were significantly associated with higher PMUM scores.

**Conclusions:**

Neurodivergence and socioeconomic disadvantage are associated with higher PMU in children, underscoring the need for routine screening and family-focused interventions in these groups.

**Disclosure of Interest:**

None Declared

